# Predicting re-fracture risk factors in older adult osteoporotic vertebral fractures patients with comorbidities: development and validation of nomogram

**DOI:** 10.3389/fmed.2025.1664157

**Published:** 2025-10-24

**Authors:** Bao Qi, Qingquan Wu, Guowu Chen, Lu Zhang, Chunyang Meng, Wei Wei, Hong Wang, Qingwei Li

**Affiliations:** ^1^Department of Spine Surgery, Affiliated Hospital of Jining Medical University, Jining, Shandong, China; ^2^Department of Interventional Radiography, Affiliated Hospital of Jining Medical University, Jining, China; ^3^Department of Medical Research Center, Affiliated Hospital of Jining Medical University, Jining, China; ^4^China Medical University, Shenyang, Liaoning, China; ^5^Department of Spine Surgery, Dalian Central Hospital, Dalian, Liaoning, China

**Keywords:** OVCF, refracture, risk factor, nomogram, PVP

## Abstract

**Background:**

Osteoporotic vertebral compression fractures (OVCFs) pose a significant health burden in older adult populations, with postoperative re-fracture (re.fra) complicating recovery. Existing models (e.g., FRAX, QFracture) inadequately address comorbidities and modifiable lifestyle factors. This study aimed to develop and validate a novel nomogram integrating these underrecognized yet critical predictors for personalized risk stratification.

**Methods:**

A retrospective cohort of 560 older adult OVCF patients undergoing percutaneous vertebroplasty (PVP) was analyzed. Patients were randomly divided into training (70%, *n* = 392) and testing (30%, *n* = 168) cohorts. Univariable and backward stepwise multivariable logistic regression identified independent re.fra predictors. A nomogram was developed and internally validated using area under the curve (AUC), calibration curves (slopes, intercepts), Brier scores, and decision curve analysis (DCA) to assess discrimination, calibration, and clinical utility.

**Results:**

Independent predictors included tumor history [adjusted odds ratio (aOR) = 12.29, 95% CI: 2.50–60.35], scoliosis (aOR = 6.46, 95% CI: 2.97–14.03), mental disorders (aOR = 5.91, 95% CI: 2.73–12.82), alcohol use ≥10 years (aOR = 3.69, 95% CI: 1.90–7.17), and chronic kidney disease (aOR = 3.12, 95% CI: 1.61–6.06). Hypertension exhibited a paradoxical protective association (aOR = 0.50, 95% CI: 0.27–0.93). The nomogram demonstrated strong discrimination [AUC:0.886 (training), 0.827 (testing)], excellent calibration in training (slope = 1.000, Brier = 0.118) with slight deviation in testing (slope = 0.697, Brier = 0.162), and superior net benefit over treat-all/none strategies across thresholds (DCA).

**Conclusion:**

This validated nomogram integrates often-overlooked comorbidities and lifestyle factors to predict post-PVP re.fra risk, providing a practical tool for personalized management and highlighting the need for multidisciplinary care in high-risk subgroups such as those with scoliosis, mental disorders, or prolonged alcohol use. The intriguing protective association of hypertension, however, requires cautious interpretation and further investigation before clinical application.

## Introduction

OVCFs pose a significant global health burden, especially in the older adult, with prevalence rising in tandem with aging populations. As the most common manifestation of osteoporotic fractures, OVCFs often lead to debilitating pain, spinal deformity, and increased mortality, profoundly impairing quality of life and imposing significant socioeconomic costs (1). PVP is a widely used minimally invasive intervention for OVCFs that do not respond to conservative treatment, providing rapid pain relief and vertebral stabilization (2). However, postoperative re.fra—occurring in up to 52% of older adult patients—remain a critical complication (3), necessitating repeat interventions and exacerbating morbidity.

Existing studies have identified demographic, and treatment-related risk factors for OVCF re.fra, including advanced age, low bone mineral density (BMD), cement leakage et al. (4, 5). However, the influence of age-related comorbidities and modifiable lifestyle factors on OVCF re.fra risk remains poorly understood. Chronic conditions such as diabetes mellitus (DM), CKD, mental disorders, and lifestyle factors like prolonged alcohol use are prevalent in older adult populations and may synergistically exacerbate skeletal fragility (6–8). Notably, prior predictive models often overlook these multifactorial interactions, relying instead on limited variables without robust validation, thereby hindering clinical utility.

This study aimed to develop and validate a nomogram that integrates both traditional and novel risk factors—including comorbidities and lifestyle variables—to predict re.fra risk in older adult OVCF patients following PVP. Using a large retrospective cohort, we identified key predictors and constructed a clinically actionable tool for personalized risk stratification. Our model emphasizes a holistic view of bone health in the older adult, with implications for international clinical practice and resource allocation.

## Methods

### Study design and cohort selection

This retrospective cohort study enrolled 560 patients diagnosed with OVCF who underwent PVP between August 1, 2015 and December 31, 2024 at a tertiary medical center. The study cohort comprised 560 patients who underwent surgical intervention for OVCF. Patients were divided into a training set (*n* = 392, 70%) and a testing set (*n* = 168, 30%) using a random sampling method to ensure balanced distribution of baseline characteristics. Inclusion criteria were: (1) age ≥50 years, (2) confirmed diagnosis of OVCF based on radiographic evidence, and (3) availability of complete clinical and follow-up data. Exclusion criteria included: (1) pathological fractures due to malignancy, (2) previous spinal surgery, and (3) incomplete medical records. This study was ethically approved by the institutional review committee of Jining Medical College (Approval No. 2024-08-C024). The studies were conducted in accordance with the local legislation and institutional requirements. The participants provided their written informed consent to participate in this study.

### Data collection and variables

Baseline demographic and clinical variables were extracted from electronic health records, including age, sex, occupation, insurance status, comorbidities [hypertension, DM, chronic obstructive pulmonary disease, CKD, mental disorders, scoliosis (defined by a Cobb angle ≥10° on standardized spinal radiographs, as confirmed by orthopedic surgeons or radiologists), and tumor history (defined as a history of benign tumors or previously treated and currently non-metastatic malignant tumors; patients with active malignancy or metastatic spinal disease were excluded as per exclusion criteria)], and lifestyle factors [alcohol use ≥10 years (defined as a history of regular alcohol consumption sustained for more than 10 years, based on retrospective electronic health record review; documented occasional or social drinking without sustained habit was excluded)]. Missing values were addressed using predictive mean matching, a multiple imputation method preserving data distribution integrity (9). OVCF re.fra was defined as a new vertebral fracture occurring within 24 months after PVP, confirmed by two independent radiologists. The follow-up period for all included patients ranged from 24 to 36 months. Variables were standardized using predefined criteria [e.g., hypertension: systolic/diastolic blood pressure ≥140/90 mmHg (10)].

### Statistical analysis

#### Baseline characteristics and group comparison

Continuous variables were reported as mean ± standard deviation and compared using Student’s t-test if normally distributed; otherwise, the Mann–Whitney *U* test was used. Normality was assessed using the Shapiro–Wilk test. Categorical variables (e.g., comorbidities) were expressed as frequencies (%) and analyzed via Pearson’s chi-square or Fisher’s exact test. A *p*-value >0.05 indicated no significant imbalance between training and testing sets, except for prespecified variables (DM, mental disorders), which were retained for adjustment in subsequent analyses.

#### Risk factor identification

Univariable logistic regression was performed to assess associations between candidate variables and OVCF re.fra. Variables with *p* < 0.10 in univariable analysis were included in a backward stepwise multivariable logistic regression model. aOR with 95% confidence intervals (CI) were computed.

#### Nomogram development

A nomogram was constructed based on the final multivariable model, with points assigned to each predictor proportional to its regression coefficient. Total points were converted to predicted OVCF re.fra probabilities using a linear predictor scale. The nomogram’s discriminative ability was visualized by mapping point ranges to probability thresholds (1–97%).

#### Model performance evaluation

Model discrimination was assessed via AUC. Calibration was evaluated using calibration curves, the Hosmer–Lemeshow test, and Brier scores (lower values indicate better accuracy). DCA quantified clinical utility by comparing net benefits across threshold probabilities (11). Since DCA can display the false- and the true-positive fractions as functions of the risk threshold, it compensates for deficiency of receiver operating characteristic (ROC) curves (12).

#### Validation strategy

Internal validation was conducted by evaluating model performance in the testing set. Overfitting was assessed by comparing training and testing AUCs, with a ΔAUC <0.10 deemed acceptable. All analyses were performed using R 4.3.0 (packages: rms, pROC, rmda), with two-tailed *p* < 0.05 considered statistically significant.

## Results

### Baseline characteristics of the cohort

The study cohort comprised 560 patients with OVCF, divided into a training set (*n* = 392, 70%) and a testing set (*n* = 168, 30%). Baseline characteristics were well-balanced between the two sets, with no significant differences in most demographic and clinical variables (all *p* > 0.05, Table 1). The mean age of the cohort was 69.91 ± 6.77 years, with a slightly higher proportion of females (54.5%) compared to males (45.5%). The majority of the patients were retirees (73.0%) and had health insurance coverage (94.8%). Key comorbidities included hypertension (62.1%), DM (42.3%), chronic obstructive pulmonary disease (54.8%), and CKD (29.5%). Notably, the prevalence of OVCF re.fra was consistent across the training (31.6%) and testing sets (38.1%, *p* = 0.166), ensuring comparable risk profiles for model development and validation. However, significant imbalances were observed in DM and mental disorders, with higher prevalence in the validation set (DM: 49.4% vs. 39.3%, *p* = 0.033; mental disorders: 29.8% vs. 21.2%, *p* = 0.038). These findings suggest that the training and validation sets were generally well-balanced for most baseline characteristics, though the observed disparities in DM and mental disorders warrant consideration in subsequent analyses to mitigate potential confounding effects.

**Table 1:** Baseline characteristics of the study population in the training and testing sets.

Clinical variables	Overall	Testing set	Training set	*p*
	*N* = 560	*N* = 168	*N* = 392	
Sex (%)				
Male	255 (45.5)	77 (45.8)	178 (45.4)	>0.999
Female	305 (54.5)	91 (54.2)	214 (54.6)	
Age (mean ± SD)	69.91 ± 6.77	69.96 ± 6.79	69.89 ± 6.77	0.909
Career (%)				
Farmer	151 (27.0)	45 (26.8)	106 (27.0)	>0.999
Retire	409 (73.0)	123 (73.2)	286 (73.0)	
Smoking_gte_10a (%)				
No	361 (64.5)	109 (64.9)	252 (64.3)	0.969
Yes	199 (35.5)	59 (35.1)	140 (35.7)	
Alcohol_gte_10a (%)				
No	376 (67.1)	112 (66.7)	264 (67.3)	0.953
Yes	184 (32.9)	56 (33.3)	128 (32.7)	
Health insurance (%)				
No	29 (5.2)	9 (5.4)	20 (5.1)	>0.999
Yes	531 (94.8)	159 (94.6)	372 (94.9)	
OP_lte_1 (%)				
No	292 (52.1)	82 (48.8)	210 (53.6)	0.346
Yes	268 (47.9)	86 (51.2)	182 (46.4)	
Hyp (%)				
No	212 (37.9)	55 (32.7)	157 (40.1)	0.124
Yes	348 (62.1)	113 (67.3)	235 (59.9)	
DM (%)				
No	323 (57.7)	85 (50.6)	238 (60.7)	0.033
Yes	237 (42.3)	83 (49.4)	154 (39.3)	
COPD (%)				
No	253 (45.2)	77 (45.8)	176 (44.9)	0.911
Yes	307 (54.8)	91 (54.2)	216 (55.1)	
ST (%)				
No	318 (56.8)	91 (54.2)	227 (57.9)	0.468
Yes	242 (43.2)	77 (45.8)	165 (42.1)	
P.ST (%)				
No	423 (75.5)	122 (72.6)	301 (76.8)	0.345
Yes	137 (24.5)	46 (27.4)	91 (23.2)	
CHD (%)				
No	316 (56.4)	90 (53.6)	226 (57.7)	0.424
Yes	244 (43.6)	78 (46.4)	166 (42.3)	
PCI (%)				
No	480 (85.7)	146 (86.9)	334 (85.2)	0.693
Yes	80 (14.3)	22 (13.1)	58 (14.8)	
Trauma (%)				
No	330 (58.9)	96 (57.1)	234 (59.7)	0.639
Yes	230 (41.1)	72 (42.9)	158 (40.3)	
Mental (%)				
No	427 (76.2)	118 (70.2)	309 (78.8)	0.038
Yes	133 (23.8)	50 (29.8)	83 (21.2)	
Ost (%)				
No	332 (59.3)	105 (62.5)	227 (57.9)	0.358
Yes	228 (40.7)	63 (37.5)	165 (42.1)	
Gout (%)				
No	535 (95.5)	162 (96.4)	373 (95.2)	0.655
Yes	25 (4.5)	6 (3.6)	19 (4.8)	
Tumor (%)				
No	542 (96.8)	162 (96.4)	380 (96.9)	0.958
Yes	18 (3.2)	6 (3.6)	12 (3.1)	
Scoliosis (%)				
No	269 (48.0)	78 (46.4)	191 (48.7)	0.685
Yes	291 (52.0)	90 (53.6)	201 (51.3)	
Operating (%)				
No	531 (94.8)	155 (92.3)	376 (95.9)	0.114
Yes	29 (5.2)	13 (7.7)	16 (4.1)	
CKD (%)				
No	395 (70.5)	118 (70.2)	277 (70.7)	>0.999
Yes	165 (29.5)	50 (29.8)	115 (29.3)	
re.fra (%)				
No	372 (66.4)	104 (61.9)	268 (68.4)	0.166
Yes	188 (33.6)	64 (38.1)	124 (31.6)	

### Independent risk factors for postoperative OVCF re.fra

Univariable and multivariable logistic regression analyses were performed to identify factors associated with OVCF re.fra. In univariable analysis, significant predictors included alcohol use for ≥10 years (OR = 2.02, 95% CI: 1.30–3.15, *p* = 0.002), hypertension (OR = 0.41, 95% CI: 0.26–0.63, *p* < 0.001), mental disorders (OR = 7.92, 95% CI: 4.63–13.55, *p* < 0.001), scoliosis (OR = 15.28, 95% CI: 8.30–28.15, *p* < 0.001), CKD (OR = 6.05, 95% CI: 3.76–9.74, *p* < 0.001), and tumor history (OR = 3.15, 95% CI: 0.98–10.12, *p* = 0.054). After adjustment in the multivariable model, alcohol use for ≥10 years (aOR = 3.69, 95% CI: 1.90–7.17, *p* < 0.001), mental disorders (aOR = 5.91, 95% CI: 2.73–12.82, *p* < 0.001), scoliosis (aOR = 6.46, 95% CI: 2.97–14.03, *p* < 0.001), CKD (aOR = 3.12, 95% CI: 1.61–6.06, *p* < 0.001), and tumor history (aOR = 12.29, 95% CI: 2.50–60.35, *p* = 0.002) remained independently associated with OVCF re.fra. Hypertension retained significance but with reduced effect size (aOR = 0.50, 95% CI: 0.27–0.93, *p* = 0.028) (Table 2).

**Table 2:** Univariate and multivariate logistic regression analysis of factors associated with risk factors for refracture.

Characteristics	No (*N* = 268)	Yes (*N* = 124)	Univariable			Multivariable		
			OR	95% CI	*p*	OR	95% CI	*p*
Sex								
Male	125 (46.6%)	53 (42.7%)						
Female	143 (53.4%)	71 (57.3%)	1.17	0.76–1.80	0.471			
Age (mean ± SD)	70.1 ± 6.5	69.5 ± 7.4	0.99	0.96–1.02	0.444			
Career								
Farmer	72 (26.9%)	34 (27.4%)						
Retire	196 (73.1%)	90 (72.6%)	0.97	0.60–1.57	0.909			
Smoking_gte_10a								
No	174 (64.9%)	78 (62.9%)						
Yes	94 (35.1%)	46 (37.1%)	1.09	0.70–1.70	0.698			
Alcohol_gte_10a								
No	194 (72.4%)	70 (56.5%)						
Yes	74 (27.6%)	54 (43.5%)	2.02	1.30–3.15	0.002	3.69	1.90–7.17	0.001
Health insurance								
No	12 (4.5%)	8 (6.5%)						
Yes	256 (95.5%)	116 (93.5%)	0.68	0.27–1.71	0.411			
OP_lte_1								
No	176 (65.7%)	34 (27.4%)						
Yes	92 (34.3%)	90 (72.6%)	5.06	3.17–8.09	0.001	1.06	0.48–2.34	0.881
Hyp								
No	89 (33.2%)	68 (54.8%)						
Yes	179 (66.8%)	56 (45.2%)	0.41	0.26–0.63	0.001	0.5	0.27–0.93	0.028
DM								
No	200 (74.6%)	38 (30.6%)						
Yes	68 (25.4%)	86 (69.4%)	6.66	4.16–10.66	0.001	1.53	0.74–3.16	0.252
COPD								
No	110 (41%)	66 (53.2%)						
Yes	158 (59%)	58 (46.8%)	0.61	0.40–0.94	0.025	0.61	0.33–1.10	0.101
ST								
No	149 (55.6%)	78 (62.9%)						
Yes	119 (44.4%)	46 (37.1%)	0.74	0.48–1.14	0.17			
P.ST								
No	224 (83.6%)	77 (62.1%)						
Yes	44 (16.4%)	47 (37.9%)	3.11	1.91–5.05	0.001	1.02	0.48–2.14	0.965
CHD								
No	189 (70.5%)	37 (29.8%)						
Yes	79 (29.5%)	87 (70.2%)	5.63	3.53–8.96	0.001	1.47	0.71–3.02	0.297
PCI								
No	232 (86.6%)	102 (82.3%)						
Yes	36 (13.4%)	22 (17.7%)	1.39	0.78–2.48	0.265			
Trauma								
No	195 (72.8%)	39 (31.5%)						
Yes	73 (27.2%)	85 (68.5%)	5.82	3.66–9.27	0.001	1.69	0.90–3.17	0.1
Mental								
No	242 (90.3%)	67 (54%)						
Yes	26 (9.7%)	57 (46%)	7.92	4.63–13.55	0.001	5.91	2.73–12.82	0.001
Ost								
No	154 (57.5%)	73 (58.9%)						
Yes	114 (42.5%)	51 (41.1%)	0.94	0.61–1.45	0.793			
Gout								
No	257 (95.9%)	116 (93.5%)						
Yes	11 (4.1%)	8 (6.5%)	1.61	0.63–4.11	0.318			
Tumor								
No	263 (98.1%)	117 (94.4%)						
Yes	5 (1.9%)	7 (5.6%)	3.15	0.98–10.12	0.054	12.29	2.50–60.35	0.002
Scoliosis								
No	177 (66%)	14 (11.3%)						
Yes	91 (34%)	110 (88.7%)	15.28	8.30–28.15	0.001	6.46	2.97–14.03	0.001
Operating								
No	258 (96.3%)	118 (95.2%)						
Yes	10 (3.7%)	6 (4.8%)	1.31	0.47–3.69	0.607			
CKD								
No	222 (82.8%)	55 (44.4%)						
Yes	46 (17.2%)	69 (55.6%)	6.05	3.76–9.74	0.001	3.12	1.61–6.06	0.001

^#^For univariate logistic regression, the *p*-value threshold was set at *p* < 0.1 for variable inclusion, while for multivariate analysis, the threshold was *p* < 0.05. ^#^The included variables were: Alcohol_gte_10a, Hyp, mental, tumor, scoliosis, and CKD.

### Nomogram for OVCF re.fra risk prediction

**Figure 1:**
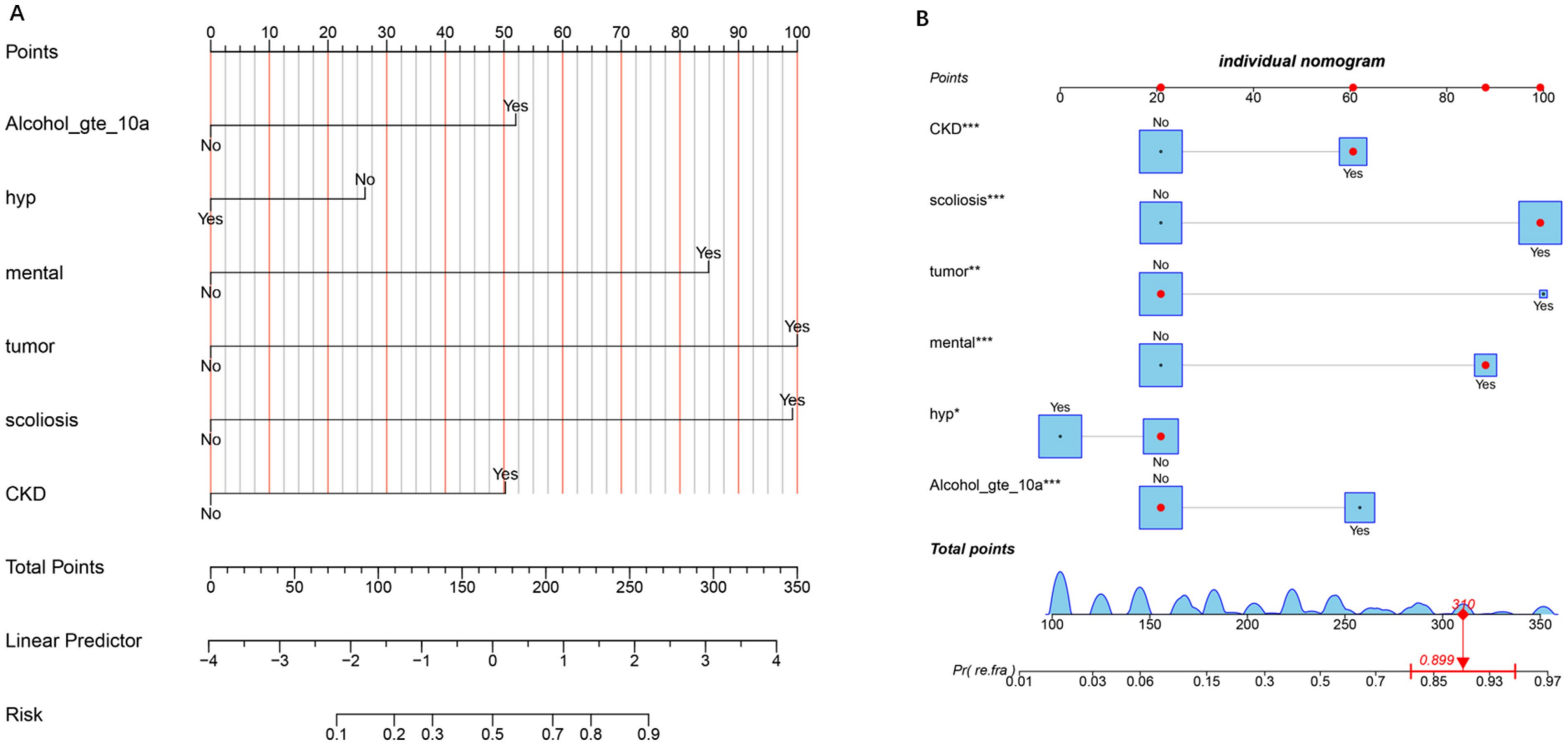
Nomogram for predicting the risk of refracture. **(A)** The nomogram illustrates the contribution of each predictor (e.g., Alcohol_gte_10a, hypertension, mental health, tumor, scoliosis, and CKD) to the total points, which are then mapped to the linear predictor and the corresponding risk probability. The “No” and “Yes” options for each predictor indicate the absence or presence of the condition, respectively. Higher total points correlate with an increased risk of the outcome. **(B)** Individual nomogram displaying the detailed point allocation for each predictor, with asterisks denoting the statistical significance of variables (^*^p < 0.05, ^**^p < 0.01, and ^***^p < 0.001). The bottom axis shows the total points, which are converted to the predicted probability of the refracture, with an example calculation (310 points ≈89.9% risk).

Nomograms for predicting OVCF re.fra risk were constructed based on the multivariable logistic regression model (Figures 1A,B). Key predictors included alcohol use for ≥10 years, hypertension, mental disorders, tumor history, scoliosis, and CKD. Each variable was assigned a weighted point score proportional to its regression coefficient. The total points derived from individual predictors were mapped to a linear predictor scale and corresponding OVCF re.fra probability. For example, a total score of 310 points translated to a predicted probability of 0.899 (89.9%). The probability scale ranged from 0.01 (1%) at 100 points to 0.97 (97%) at 350 points, demonstrating the model’s discriminative capacity across a wide risk spectrum.

### Predictive performance of the model

**Figure 2:**
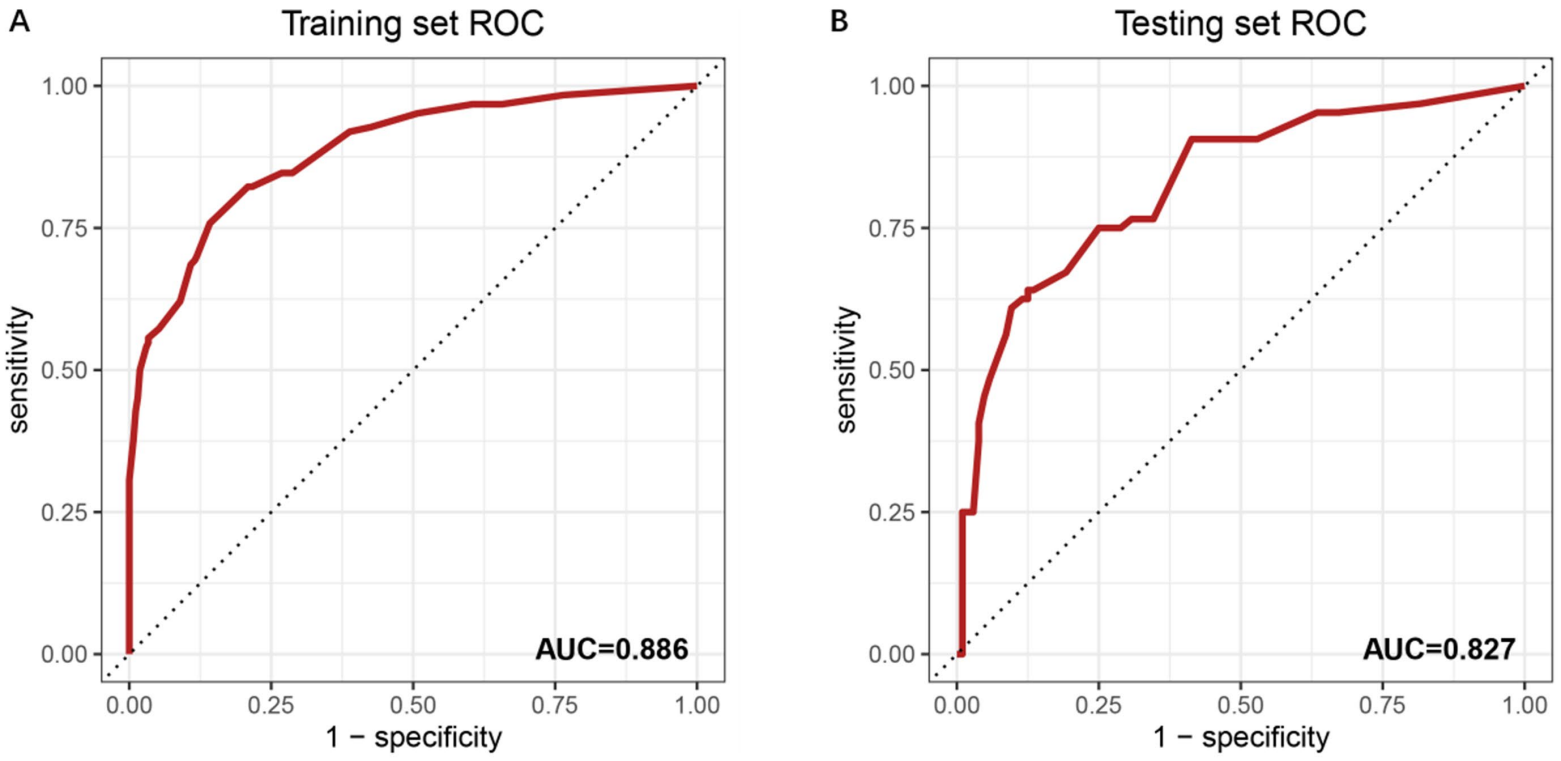
ROC curves for model performance evaluation. **(A)** ROC curve of the training set with an AUC of 0.886. The *x*-axis represents the false positive rate (1-specificity), and the *y*-axis denotes the true positive rate (sensitivity). The diagonal dashed line indicates a reference performance (AUC = 0.5). Numerical labels (0.00, 0.25, 0.50, 0.75, 1) correspond to key thresholds for specificity and sensitivity. **(B)** ROC curve of the independent testing set, showing an AUC of 0.827. The reduced AUC compared to the training set suggests the model’s generalizability. Threshold values (0.00, 0.25, 0.50, 0.75, 1) align with standard ROC interpretation.

The predictive performance of the model was evaluated using ROC curve analysis for both the training and testing datasets (Figures 2A,B). In the training set, the model demonstrated excellent discriminative ability, with an AUC of 0.886. This high AUC value indicates strong predictive accuracy in distinguishing between individuals with and without OVCF re.fra within the training cohort. The model’s performance was further validated in the testing set, where it achieved an AUC of 0.827. Although slightly lower than the training set, this AUC value still reflects good predictive performance, suggesting that the model generalizes well to the unknown data. The minimal reduction in performance (ΔAUC = 0.059) implies no substantial overfitting, underscoring the model’s stability and clinical applicability.

### Calibration of the prediction model

**Figure 3:**
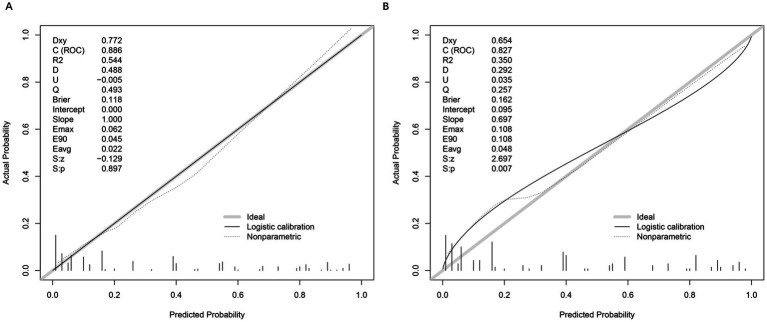
Calibration plots and performance metrics for model validation. **(A)** Calibration plot of the training set, comparing actual versus predicted probabilities. Key metrics include Somers’ *D_xy_* (0.772), *C*-index (ROC = 0.886), *R*^2^ (0.544), and Brier score (0.118). The logistic calibration curve (solid line) and nonparametric ideal line (dashed) demonstrate model fit. Slope (1.000) and intercept (0.000) indicate minimal calibration drift. Additional metrics (*E*_max_ = 0.062, *E*_90_ = 0.045, *E*_avg_ = 0.022) reflect small calibration errors. **(B)** Moderate agreement between predicted and observed OVCF re.fra probabilities, with a calibration slope of 0.697 (ideal = 1.000), Brier score of 0.162, and integrated calibration index (*E*_avg_ = 0.048), reflecting an average 4.8% deviation between predictions and outcomes. While the model retained clinical utility (Brier <0.2), significant slope deviation (0.697 vs. 1.0) and intercept shift (0.095) indicated overfitting and systematic overestimation in high-risk subgroups (predicted probabilities >0.3), as evidenced by nonparametric calibration divergence and elevated errors (*E*_max_ = *E*_90_ = 0.108, *E*_avg_ = 0.048). The Spiegelhalter test (S: *p* = 0.007) and reduced discrimination metrics (*D_xy_* = 0.654, *C*-index = 0.827, *R*^2^ = 0.350) further underscored the need for recalibration to improve accuracy in high-risk populations. These deviations primarily occur in two regions: (1) For predicted probabilities <0.3, the model shows slight overestimation of risk (observed events were ~10% lower than predicted), likely due to fewer low-risk cases in our cohort; (2) At higher predicted probabilities (0.7–0.9), we observe modest underestimation, where actual event rates exceeded predictions by ~8%.

The calibration of the prediction model was assessed using calibration curves for both the training and testing sets (Figures 3A,B). In the training set, the model demonstrated excellent calibration, with a calibration slope of 1.000 and an intercept of 0.000, indicating near-perfect agreement between predicted and observed probabilities. The Brier score, a measure of overall model accuracy, was 0.118, further supporting the model’s strong predictive performance. In the testing set, the model maintained good calibration, though with a slight decrease in performance compared to the training set. The calibration slope was 0.697, and the intercept was 0.095, suggesting minor deviations from ideal calibration. The Brier score increased to 0.162, reflecting a modest reduction in accuracy. Despite this, the model retained strong discriminatory power and reasonable calibration in the independent validation cohort.

**Figure 4:**
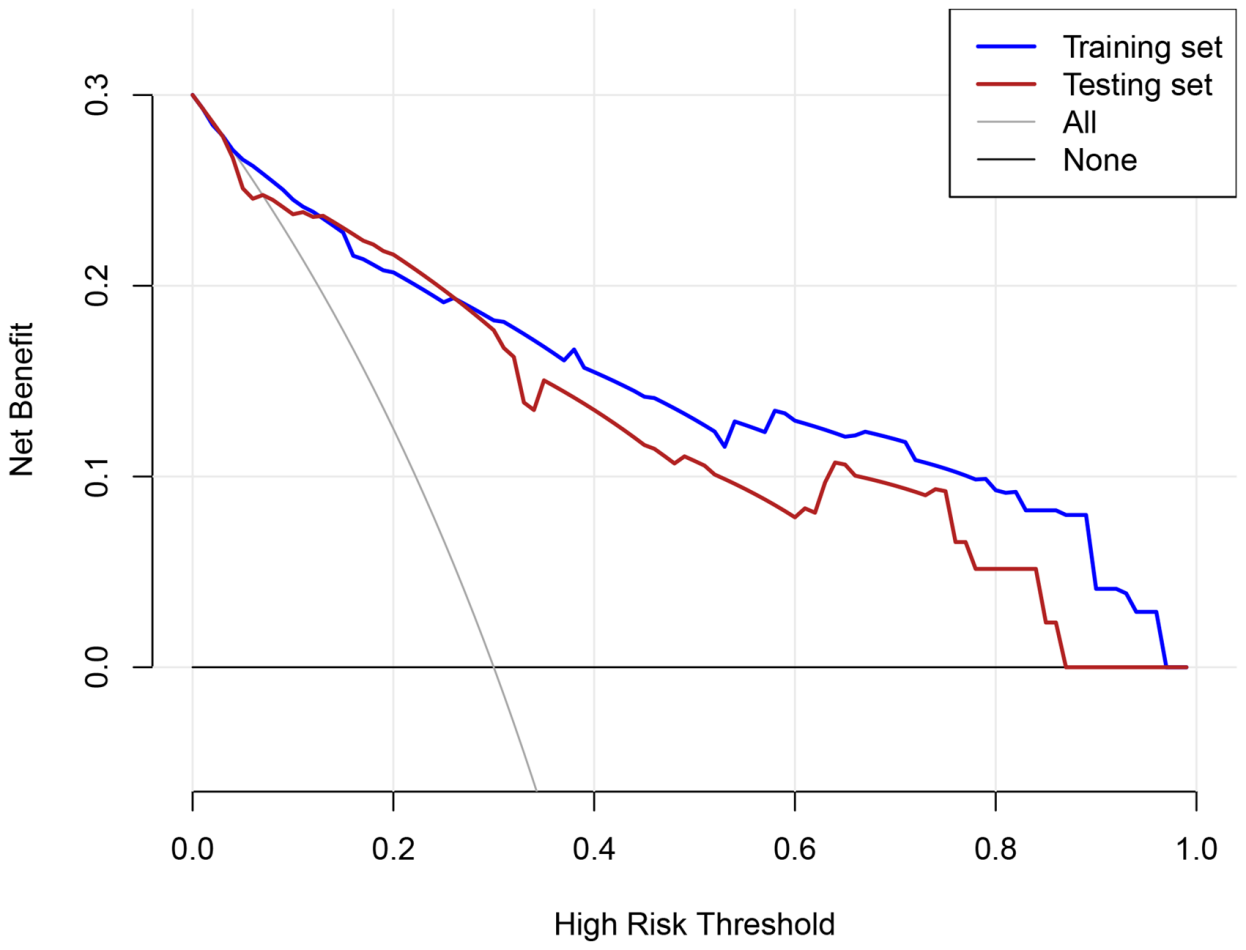
DCA evaluating the clinical utility of the predictive model across different risk thresholds.

These patterns may reflect the testing set’s higher prevalence of diabetes (49.4% vs. 39.3%) and mental disorders (29.8% vs. 21.2%), which could amplify risk in high-risk subgroups. Importantly, despite these calibration deviations, the model maintains strong discriminative ability (AUC = 0.827) and clinical utility across all thresholds (DCA in Figure 4), suggesting limited practical impact on risk stratification.

### Clinical utility of the prediction model

The clinical utility of the prediction model was evaluated using DCA across the training and testing sets (Figure 4). In the training set, the model demonstrated a consistently higher net benefit compared to the “treat all” and “treat none” strategies across most threshold probabilities, particularly in the range of 0.1 to 0.8. Similarly, in the testing set, the model maintained a higher net benefit over a wide range of thresholds, although the net benefit was slightly lower compared to the training set. The DCA highlights that the prediction model provides significant clinical value across a broad spectrum of risk thresholds, enabling clinicians to tailor interventions based on individualized risk assessments.

The model demonstrated strong discriminative ability in both cohorts, with an area under the curve (AUC) of 0.886 (95% CI: 0.850–0.922) in the training cohort and 0.827 (95% CI: 0.762–0.893) in the validation cohort. Further evaluation through decision curve analysis, plotting net benefit for the training set (solid line), testing set (dashed line), and reference strategies (“All” and “None”), revealed superior clinical utility across the 0.1–1.0 risk threshold range. This confirms its potential for guiding clinical decisions within clinically relevant probability thresholds.

## Discussion

This study developed and validated a nomogram to predict postoperative re.fra risk in patients with OVCF undergoing surgical intervention. By analyzing a cohort of 560 patients, we identified several independent risk factors, including alcohol use ≥10 years, mental disorders, scoliosis, CKD, and tumor history. The model demonstrated strong discriminative performance (AUC: 0.886 in training, 0.827 in testing), good calibration, and significant clinical utility across a wide range of risk thresholds. These findings underscore the model’s potential to guide personalized postoperative management and preventive strategies in high-risk populations.

### The rationality and robustness of Nomotus construction

Our nomogram was developed using established clinical prediction model methods, incorporating multivariable logistic regression to combine independent predictors into a user-friendly visual tool. This approach is supported by prior studies demonstrating the utility of nomograms in refracture risk prediction (13, 14). Our model incorporates six clinically significant predictors, including both modifiable (e.g., alcohol use) and non-modifiable (e.g., scoliosis) factors, aligning with previous research highlighting their roles in bone health and fracture risk (15). The inclusion of novel factors such as mental disorders and tumor history, alongside traditional variables like CKD and scoliosis, enhances the model’s specificity and predictive granularity, addressing a critical gap in existing tools that often overlook these factors. The nomogram’s robust performance is evidenced by high AUC values (0.886 in the training set and 0.827 in the testing set), acceptable calibration (Brier scores: 0.118–0.162), and minimal overfitting (ΔAUC = 0.059), which are comparable to or exceed those of widely used models like fracture risk assessment tool (16) and QFracture (17). These metrics underscore the model’s generalizability and clinical applicability, consistent with guidelines for transparent and reproducible predictive modeling (18). By translating complex statistical outputs into actionable risk probabilities, this nomogram provides a practical tool for tailored interventions in patients post-PVP.

### Interpretation of key risk factors

This study identified a history of neoplasms as a critical risk factor for OVCF re.fra, demonstrating an exceptionally high aOR of 12.29 (95% CI: 2.50–60.35, *p* = 0.002). Probably involves the following mechanisms: (1) direct tumor-mediated bone destruction through receptor activator of NF-κB ligand/osteoprotegerin axis dysregulation, increasing osteoclast activity and (2) treatment-induced skeletal damage, where Chemotherapy can be affected on physiological function of movement system and the skeleton construction. Mineral status disorders and skeletal changes lead to secondary forms of osteopenia and osteoporosis which would increase the risk of fracture (19, 20). However, the limited subgroup sizes (*n* = 18 with tumors). Future studies should prioritize multicenter cohorts to expand tumor subgroup analyses, integrate longitudinal receptor activator of nuclear factor-kappa B ligand/osteoprotegerin monitoring with imaging, elucidate chemotherapy-induced osteotoxicity mechanisms, and test multimodal interventions (antiresorptives, tailored exercise, nutritional support) for fracture prevention in cancer survivors. Clinical protocols for post-PVP management must prioritize cancer survivors, implementing enhanced monitoring (biannual DXA with trabecular bone score) and early antiresorptive therapy [zoledronic acid reduced fracture risk by 10% in this subgroup (21)]. These findings underscore the need for oncology-orthopedics collaborative care models to address this high-risk population.

This study identified scoliosis as the most significant risk factor for OVCF re.fra except tumor history (aOR = 6.46, 95% CI: 2.97–14.03, *p* < 0.001), exerting the profound impact through altered spinal biomechanics and uneven load distribution. The previous biomechanical studies emphasizing scoliosis as a critical important of vertebral stress redistribution (22). Scoliosis alters load distribution across adjacent vertebrae, increasing fracture susceptibility—a mechanism corroborated by Fang et al. (23), who reported scoliosis is an independent risk factor for re.fra after OVCF laminoplasty and a possible risk factor for re.fra after surgery. In osteoporotic patients, this mechanical instability is further exacerbated by reduced BMD, creating a synergistic risk environment (24, 25). The inclusion of scoliosis in our nomogram provides a critical tool for identifying high-risk individuals, particularly those with severe degenerative scoliosis, who may benefit from targeted interventions. These interventions include bracing to redistribute spinal loads, physical therapy, and early osteoporosis prevention measures. Additionally, addressing malnutrition, which is common in the older adult and impairs bone healing, through adequate intake of protein, calcium, and vitamin D is crucial for bone health and fracture prevention (26).

Mental disorders merged as significant independent risk factors for OVCF re.fra, demonstrating a nearly six-fold increased risk (aOR = 5.91, 95% CI: 2.73–12.82, *p* < 0.001). This finding aligns with comprehensive meta-analyses indicating 51% higher fracture rates among psychiatric patients compared to the general population (27). Probably involves the following mechanisms: (1) pharmacological effects of psychotropic medications, particularly selective serotonin reuptake inhibitors, with longitudinal studies demonstrating 4–6% BMD reduction through anticholinergic-mediated osteoclast activation (28). (2) Behavioral consequences of mental illness, including poor adherence to anti-osteoporosis therapies and sedentary lifestyles, directly impair bone remodeling capacity (29). (3) Malnutrition and depression-induced endocrine alterations (depression is associated with decreased levels of gonadal hormones estrogen and testosterone, which are key regulators of bone formation) create a catabolic metabolic environment (28, 29). The high prevalence of mental disorders in our cohort (23.8%) underscores the critical need for integrated care models that simultaneously address psychiatric and bone health. Such models should incorporate routine bone density monitoring, fall prevention strategies, and medication reviews for patients receiving long-term psychotropic treatment. These findings emphasize the importance of multidisciplinary approaches in OVCF management to mitigate the substantial re.fra risk associated with mental health comorbidities.

This study identified prolonged alcohol consumption as a significant modifiable risk factor for OVCF re.fra, with an aOR of 3.69 (95% CI: 1.90–7.17, *p* < 0.001). Probably involves the following mechanisms: chronic alcohol consumption exerts direct toxic effects on osteoblasts, suppressing bone formation as evidenced by reduced serum osteocalcin levels and histomorphometric findings of decreased trabecular bone volume and osteoid synthesis (30). Concurrently, alcohol disrupts calcium-regulating hormones: acute intoxication induces transient hypoparathyroidism, leading to hypocalcemia and hypercalciuria, while chronic abuse is associated with impaired vitamin D metabolism, including reduced serum levels of 25-hydroxyvitamin D and 1,25-dihydroxyvitamin D. This results in diminished intestinal calcium absorption and compensatory secondary hyperparathyroidism, which fails to adequately stimulate bone remodeling due to alcohol-induced skeletal resistance to parathyroid hormone. Additionally, malnutrition (e.g., low dietary calcium and protein intake), magnesium deficiency, and liver dysfunction exacerbate these effects by further impairing vitamin D activation, calcium homeostasis, and osteoblast function (31). Collectively, these pathways culminate in a low bone turnover state characterized by reduced bone formation and accelerated skeletal fragility. The elevated aOR in our surgical cohort compared to population-based studies (OR = 1.5–2.0) likely reflects synergistic interactions with perioperative risk factors. These findings emphasize the critical need for structured alcohol cessation programs in post-PVP care, particularly for patients with >10-year consumption history. It is worth mentioning that data on variables such as alcohol consumption were retrospectively collected from medical records, which may lack precise quantitative details. Therefore, future prospective studies will benefit from standardized tools that more accurately quantify alcohol intake.

Previous research has established complex associations between hypertension and bone metabolism, with multiple studies demonstrating a positive correlation between hypertension and lumbar spine BMD, however the results are conflicting (32). Epidemiological evidence suggests hypertensive patients face increased osteoporosis risk, potentially due to a similar pathogenetic etiology between hypertension and osteoporosis (33). However, our study revealed a paradoxical protective association between hypertension and OVCF re.fra risk (aOR = 0.50, 95% CI: 0.27–0.93, *p* = 0.028), potentially mediated through antihypertensive pharmacotherapy. This apparent contradiction may be explained by specific therapeutic interventions: thiazide diuretics demonstrate bone-protective effects through enhanced calcium homeostasis, showing 6.03% higher lumbar spine BMD in users compared to non-users (34), while calcium channel blockers may directly stimulate osteoblast activity (35). Furthermore, a bone metabolic mechanisms study demonstrat that thiazide diuretics’ direct osteoanabolic effects through NCC expression in osteoblasts, enhancing differentiation via increased Runx2/osteopontin expression and mineralized nodule formation (36). Nevertheless, this protective association should be interpreted with caution, as it may also stem from unmeasured confounders such as nutritional factors or vitamin D status, which were not fully adjusted for in our analysis. The potential mediating role of specific antihypertensive agents—particularly the purported skeletal benefits of thiazide diuretics—remains a compelling yet unverified hypothesis. Future studies should incorporate prospectively collected medication data to evaluate class-specific effects and include longitudinal biomarkers to disentangle direct and pharmacologically mediated effects. Until then, despite this observed association, clinical practice should maintain standard osteoporosis management for all OVCF patients, regardless of hypertension status.

### Limitations

This study has several limitations: (1) As a single-center, retrospective study with only internal validation, the generalizability of the nomogram may be limited by regional variations in patient demographics, clinical practices, and healthcare systems; (2) the small sample size in certain subgroups—particularly tumor history (*n* = 18)—may lead to statistical instability and overestimation of effects, and unmeasured confounders such as nutritional status, medication adherence, and vitamin D levels might further influence refracture risk; these findings thus require cautious interpretation; (3) The model was developed and validated in a specific Chinese population, and its performance may be influenced by genetic, lifestyle, dietary, or medical system differences in other regions. Thus, external validation in diverse international cohorts is essential before broader application.

## Conclusion

This study developed and validated a clinically practical nomogram for predicting re.fra risk in older adult OVCF patients undergoing PVP, integrating both traditional and novel risk factors such as umor history, scoliosis, mental disorders, prolonged alcohol use and CKD. The model demonstrated robust discriminative performance, excellent calibration, and significant clinical utility across diverse risk thresholds. These findings highlight the critical interplay between comorbidities, lifestyle factors, and bone health, providing a tailored tool for risk stratification and personalized postoperative management. Future multicenter studies should further validate these predictors and explore targeted interventions to mitigate re.fra in high-risk populations.

## Data Availability

The original contributions presented in the study are included in the article/supplementary material, further inquiries can be directed to the corresponding authors.
